# The Critical Assessment of Small Molecule Identification (CASMI): Challenges Solutions

**DOI:** 10.3390/metabo3030517

**Published:** 2013-06-25

**Authors:** Emma L. Schymanski, Steffen Neumann

**Affiliations:** 1 Eawag: Swiss Federal Institute of Aquatic Science and Technology, Überlandstrasse 133, Dübendorf CH-8600, Switzerland; 2 IPB: Leibniz Institute of Plant Biochemistry, Department of Stress and Developmental Biology, Weinberg 3, Halle (Saale) DE-06120, Germany

**Keywords:** mass spectrometry, metabolite identification, small molecule identification, contest, metabolomics, non-target identification

## Abstract

The *C*ritical *A*ssessment of *S*mall *M*olecule *I*dentification, or CASMI, contest was founded in 2012 to provide scientists with a common open dataset to evaluate their identification methods. In this article, the challenges and solutions for the inaugural CASMI 2012 are presented. The contest was split into four categories corresponding with tasks to determine molecular formula and molecular structure, each from two measurement types, liquid chromatography-high resolution mass spectrometry (LC-HRMS), where preference was given to high mass accuracy data, and gas chromatography-electron impact-mass spectrometry (GC-MS), *i.e.*, unit accuracy data. These challenges were obtained from plant material, environmental samples and reference standards. It was surprisingly difficult to obtain data suitable for a contest, especially for GC-MS data where existing databases are very large. The level of difficulty of the challenges is thus quite varied. In this article, the challenges and the answers are discussed, and recommendations for challenge selection in subsequent CASMI contests are given.

## 1. Introduction

The CASMI contest, the *C*ritical *A*ssessment of *S*mall *M*olecule *I*dentification, was founded in 2012. The aim of CASMI [1] was to encourage experts to exhibit their identification methods on a common dataset and, thus, enable a better comparison of the methods available. The task was to determine the molecular formula and/or the molecular structure from the mass spectrometry data. The myriad of options available for small molecule identification (vendor software, specialized independent software, open access and open source options) makes it increasingly difficult for users and researchers alike to keep pace with the changes. Instead, offering a common dataset enables the use of expert knowledge or any chosen identification methods and provides a basis for comparison. The aim of CASMI was to include all disciplines interested in small molecule identification and, thus, enable the cross-disciplinary exchange of information and expertise. In this article, small molecule identification refers to molecules of approximately 50-1,000 Da that can be detected with mass spectrometric (MS) techniques. 

Although MS identification methods are often categorized according to the chromatographic separation used (e.g., gas chromatography (GC) *versus* liquid chromatography (LC)), with relatively recent instrumental developments, such as high resolution and soft-ionization GC-MS, it is difficult to distinguish separation, detection and identification techniques and set distinct categories for a competition to allow a broad range of participants. The inaugural CASMI focused on two measurement types, liquid chromatography-high resolution mass spectrometry (LC-HRMS), where preference was given to high mass accuracy MS/MS data, and gas chromatography-electron impact-mass spectrometry (GC-MS), focusing on unit mass accuracy data. Although this excluded some participants, e.g., those with only unit mass accuracy LC-MS/MS data experience and those with high mass accuracy GC-MS data, these categories could be considered for future CASMI contests. 

The data collection commenced in the early months of 2012, with the original aim of 20 challenges per category. The ‘unknowns’ could not be truly unknown for the purpose of the competition and, thus, required a confirmed identity. However, this made it difficult to obtain suitable data, especially for the GC-MS data where many of the challenges available were also in common databases. In the end, challenges were obtained from plant material, environmental samples and reference standards. As it was difficult to find GC-MS challenges that were not in the NIST database [2], challenges were provided that only had a relatively low probability (≤60%) in the database search, although a couple with high probability (>90%) were added to give some variety. This compromise meant that the level of difficulty of the challenges was quite varied. Despite this compromise, however, the initial target of 20 suitable challenges for each category was not achieved. In the GC-MS dataset, the final 16 challenges were all confirmed with reference standards, and although other substances were available in the samples provided, they did not have matching standards. For the LC-HRMS data, it was difficult to obtain identified “unknowns”, as those already published could be linked to the names and/or institutes of the organizers, while those unpublished were often intended for a forum other than CASMI. This finally resulted in 14 LC-HRMS challenges. Six of the challenges were part of pathway elucidation efforts to determine gene function during investigations into the biochemistry of natural products and their role in the development and defenses of plants. The remaining eight environmental substances were taken from “failed confirmations” (which were not suitable for publication alone) and one successful target identification of a rare compound, not yet published. 

In retrospect, given the small number of participants for the first CASMI and the fact that not all participants contributed to all challenges within a category, the number of challenges seemed appropriate. Although a smaller number of challenges may have encouraged more participants, a larger number of challenges is needed to provide sufficient variety in the difficulty and chemical diversity of the challenges and to allow a proper evaluation. The disadvantage of providing many challenges is that it creates an advantage for fully automated entries, as methods requiring the input of human expertise are generally more time-consuming. 

In this article, the challenges and the answers are discussed, along with recommendations for challenge selection in subsequent CASMI contests. Details about the participants and the outcome of the first contest can be found in [3], also in this special issue. 

## 2. LC-HRMS Challenges and Solutions (Category 1 and 2)

The LC-HRMS challenges were sourced from plant material and standards purchased for confirmation of unknowns in environmental investigations. All challenges contained the elements C, H, N, O, P and S; no halogens were present in any compound; see Table 1 for an overview. Appendix A contains annotated spectra for each challenge, which show the composite spectrum of all available MS/MS files for each challenge and, thus, display the most intense peak where a given peak occurred in multiple spectra within the error window of 0.0001 Da plus 5 ppm. The MS spectra were also included for certain challenges. The fragments were annotated using ACD ChemSketch [4] and Mass Frontier [5] and processed automatically using OpenBabel [6] and a script in R [7] to determine placement. Although the most realistic fragments were selected, many of these are tentative and have not been confirmed unambiguously. Appendix B provides more details on the challenge compounds, including PubChem and ChemSpider identifiers. 

As it proved difficult to obtain suitable challenge compounds for this contest, there was no ‘easy *vs.* hard’ pre-selection. In the end, this meant some of the challenges were quite challenging, while others were too easy. Although challenges that were in reference databases were avoided as far as possible, some compounds were uploaded to MassBank [8] after the challenge data was released. 

### 2.1. LC-HRMS Challenges 1 to 6

The first six challenges were metabolites that were encountered as part of plant metabolomics research. The compounds were measured on a Bruker micrOTOF-Q equipped with an electrospray ionization (ESI) source in positive mode, which generally achieves ≤5 ppm mass accuracy and 12,000 resolution during routine measurements. At this resolution, the extraction of the isotopic fine structure (which would resolve, e.g., the ^15^N or ^34^S isotope peaks) is not possible, but the isotope intensities are generally very accurate. The data was acquired with a 3 Hz scan frequency for both MS and MS/MS acquisitions. 

**Table 1:** Liquid chromatography (LC) Challenges for the *C*ritical *A*ssessment of *S*mall *M*olecule *I*dentification (CASMI) 2012.

Challenge	Trivial Name	Formula	Exact mass
1	Kanamycin A	C_18_H_36_N_4_O_11_	484.2381
2	1,2-Bis-O-sinapoyl-beta-D-glucoside	C_28_H_32_O_14_	592.1792
3	Glucolesquerellin	C_14_H_27_NO_9_S_3_	449.0848
4	Escholtzine	C_19_H_17_NO_4_	323.1158
5	Reticuline	C_19_H_23_NO_4_	329.1627
6	Rheadine	C_21_H_21_NO_6_	383.1369
10	1-Aminoanthraquinone	C_14_H_9_NO_2_	223.0633
11	1-Pyrenemethanol	C_17_H_12_O	232.0888
12	alpha-(o-Nitro-p-tolylazo)acetoacetanilide	C_17_H_16_N_4_O_4_	340.1172
13	Benzyldiphenylphosphine oxide	C_19_H_17_OP	292.1017
14	1H-Benz[g]indole	C_12_H_9_N	167.0735
15	1-Isopropyl-5-methyl-1H-indole-2,3-dione	C_12_H_13_NO_2_	203.0946
16	[1-(4-methoxyanilino)-1-oxopropan-2-yl] 6-oxo-1-propylpyridazine-3-carboxylate	C_18_H_21_N_3_O_5_	359.1481
17	Nitrin	C_13_H_13_N_3_	211.1109

**Figure A1:**
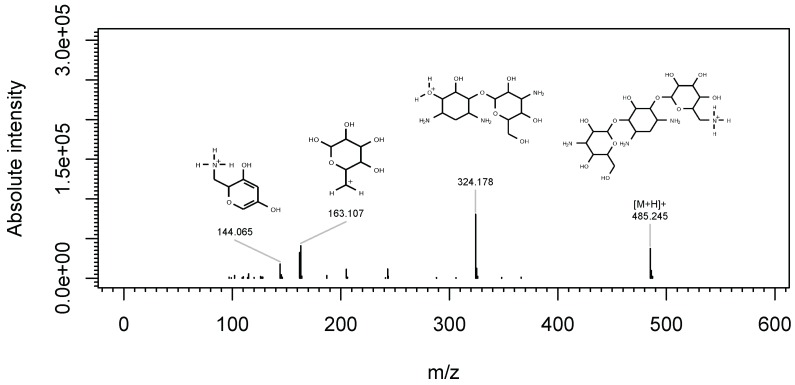
Challenge 1: annotated merged MS and MS/MS spectra of kanamycin A (electrospray ionization (ESI), positive mode).

**Challenge 1** was kanamycin A (C_18_H_36_N_4_O_11_), an aminoglycoside compound with antibiotic effects from bacteria. The compound was available as an authentic standard. The challenge data comprised the full-scan data, including two isotope peaks and fragment-rich MS/MS spectra at 10 eV, 20 eV and 30 eV in positive mode, shown in Figure A1. The MS/MS of the three collision energies were acquired in consecutive scans, which reduced the effective scan frequency for one collision energy to 1 Hz. The LC-HRMS/MS data was processed with the XCMS centWave feature detection [9], and the compound spectrum was extracted with CAMERA [10]. This approach is described in greater detail in [11]. 

**Figure A2:**
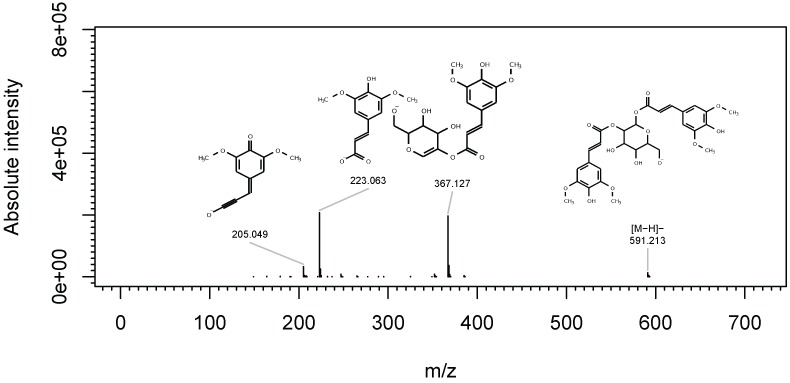
Challenge 2: annotated MS/MS spectrum of 1,2-bis-O-*β*-sinapoyl-D-glucoside (ESI, negative mode).

**Challenge 2** was 1,2-bis-O-sinapoyl-*β*-D-glucoside (C_28_H_32_O_14_), which was extracted from canola seeds and characterized previously [12]. The challenge data in negative mode included isotopes up to (M + 3) and a single fragment-rich MS/MS spectrum, shown in Figure A2, which was also extracted with XCMS and CAMERA, as described above. The raw data provided initially was affected by a severe calibration problem, resulting in ≈30 ppm mass deviation. The data, recalibrated to within 5 ppm accuracy, was provided to the participants after the contest closed, to offer them a chance to recalculate their results on more accurate data for the special issue. 

**Figure A3:**
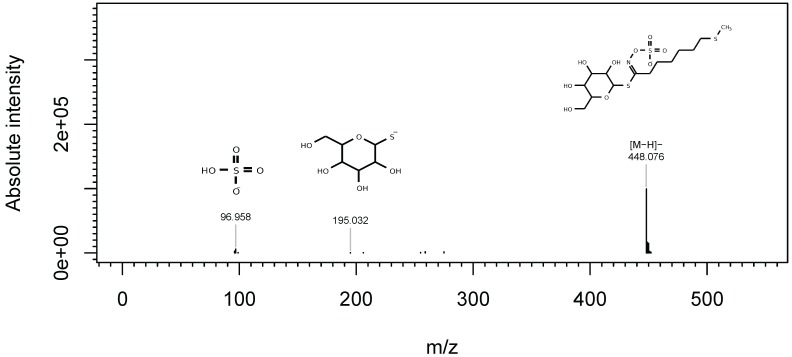
Challenge 3: annotated merged MS/MS spectra of glucolesquerellin (ESI, negative mode).

**Challenge 3** was glucolesquerellin (C_14_H_27_NO_9_S_3_), found with other glucosinolates in the seeds of *Brassicacae*. Among others, the glucosinolates 3-methylthiopropyl (3MTP, glucoibervirin), 4-methylthiobutyl (4MTB, glucoerucin), 7-methylthioheptyl (7MTH) and 8-methylthiooctyl (8MTO) are described in [13]. The challenge data was measured from a methanolic extract of *Arabidopsis thaliana* seeds, in negative mode. Although no authentic standard was used, the confidence in the identification was quite high based on the molecular formula determined with high mass accuracy data, characteristic product ions and the consistency of the structural information (including retention time) with other glucosinolates of different chain lengths. Isotopes were present up to (M + 4). The MS/MS spectra (see Figure A3) were extracted with XCMS and CAMERA (as described above) and did not contain the precursor ion for collision energies above 20 eV. 

**Figure A4:**
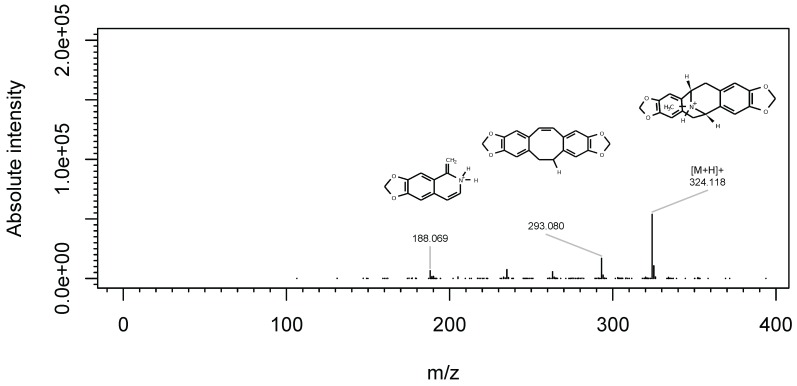
Challenge 4: annotated merged MS/MS spectra of escholtzine (ESI, positive mode).

**Figure A5:**
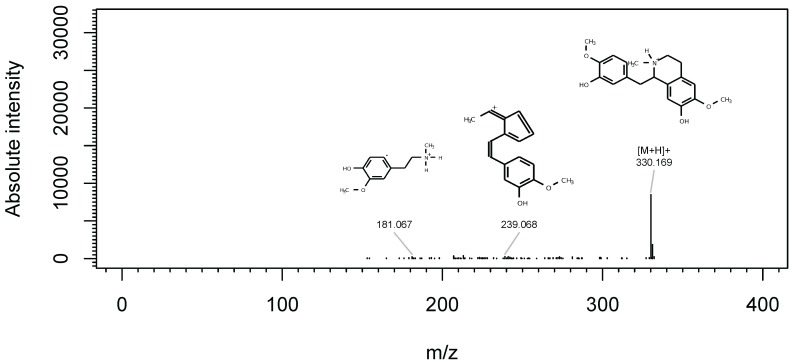
Challenge 5: annotated merged MS/MS spectra of reticuline (ESI, positive mode).

**Figure A6:**
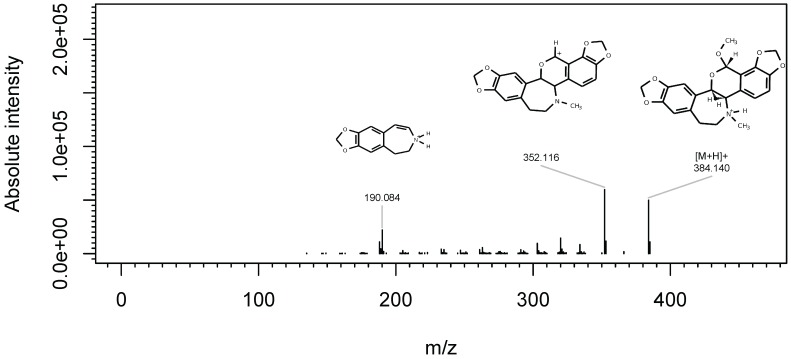
Challenge 5: Challenge 6: annotated mergedMS/MS spectra of rheadine (ESI, positive mode).

**Challenges 4-6** were combined into a single sample and measured together in positive mode. As for Challenge 1, the collision energy was alternated in the raw file, but in contrast to the previous challenges, the MS/MS data was extracted from a single scan for each compound and collision energy. All peaks below an intensity of 1% of the base peak were removed. The spectra are given in Figure A4, Figure A5 and Figure A6. The data provided originally was not calibrated and had mass deviations up to 8 ppm. After the closing of the contest, the data was recalibrated and provided to the participants. This resulted in deviations below 5 ppm for Challenges 4 and 6, but at the same time, increased the mass error for Challenge 5 to approximately 6 ppm. 

Challenge 4 was the alkaloid escholtzine (C_19_H_17_NO_4_). The isotopic pattern included only peaks up to (M + 2), while the 30 eV MS/MS spectrum was very noisy. 

Challenge 5 was another alkaloid, reticuline (C_19_H_23_NO_4_). While the 20 eV MS/MS spectrum still contains the precursor, the 30 eV spectrum contains a few additional fragments below *m*/*z* 176. 

Challenge 6 was the alkaloid rheadine (C_21_H_21_NO_6_). The MS/MS spectra contained more fragments than the previous challenge. 

As all of these compounds were in PubChem, they could be considered “known unknowns”. Challenges 7 to 9 are absent; as discussed above, the original aim of 20 challenges was not attained, and the original numbering was kept in this article for consistency with the participant results and publications. 

### 2.2. LC-HRMS Challenges 10 to 17

These challenges resulted from unconfirmed tentative identifications arising from the effect-directed analysis (EDA) of river water sampled from the Elbe (Czech Republic) using the passive sampler, blue rayon [14], where CASMI provided some ‘use’ for standards that otherwise had no specific purpose. As a result, some of these are quite challenging challenges, whereas others are more straightforward. All these challenges were taken from measurements of reference standards, using either ESI or atmospheric pressure chemical ionization (APCI) techniques; the fragmentation modes were either collision-induced dissociation (CID) or higher-energy collisional dissociation (HCD); the settings reported are as normalized collision energies (NCE). 

**Challenge 10** was 1-aminoanthraquinone, shown in Figure A. Although amino groups are usually a distinctive loss in many compounds, here, the first losses are a water from a carbonyl group (*m*/*z* 206), resulting in a rearrangement to form a stabilizing four-membered ring with the amino-substituent, as well as the loss of the full carbonyl group itself (*m*/*z* 196). The loss of a full benzyl group results in the fragment at *m*/*z* 146, likely also stabilized by the formation of a four-membered ring; while the remaining fragment at *m*/*z* 105.033 is likely to result from the loss of the same benzyl ring along with one of the carbonyl groups, where the charge remains with the smaller fragment. The accurate mass of the fragment confirms the formula C_7_H_5_O, rather than, e.g., a nitrogen adduct (*m*/*z* 105.044), such as those seen in [15]. 

**Figure A8:**
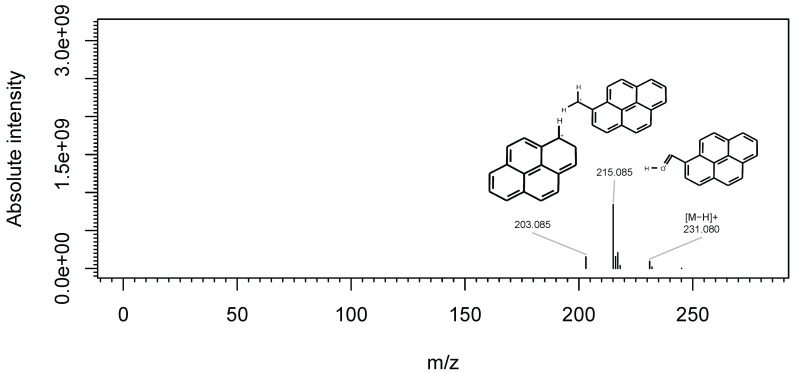
Challenge 11: annotated merged MS and MS/MS spectra of 1-pyrenemethanol (APCI, positive mode).

**Challenge 11** was 1-pyrenemethanol and had a difficult MS spectrum to interpret, although the very simple fragmentation pattern was also informative. Both the MS and MS/MS are plotted in Figure A8. The behavior of substances can be a lot less consistent with APCI and atmospheric pressure photo ionization (APPI) compared with ESI, and this substance undergoes an in-source loss and oxidation to an [M − H]^+^ ion. The only losses are the hydroxy group and the complete methanol substituent. The fact that no other fragments are generated despite targeted MS/MS on the *m*/*z* 215 peak indicates that a stable aromatic backbone is likely to be present. In-source oxidation has been reported previously, for example in [16], the isobars, tonalide and galaxolide, could not be separated chromatographically, but could be identified using their different ionization behavior in positive mode. Tonalide was visible as both [M]^+^^·^ and [M + H]^+^, whereas galaxolide was detected as [M − H]^+^ (an in-source oxidation product) and the [M]^+^^·^ ion. The authors explained this with differing proton affinities, demonstrating that galaxolide has a lower proton affinity than the proton donors in the APPI source and, thus, competed unfavorably for the protons. 

**Figure A9:**
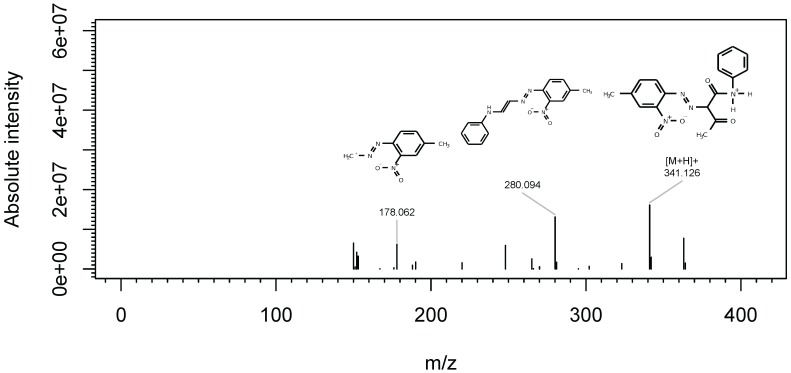
Challenge 12: annotated merged MS and MS/MS spectra of “Pigment Yellow 1” (APCI, positive mode).

**Challenge 12** was *α*-(o-nitro-p-tolylazo)acetoacetanilide, commonly known as “Pigment Yellow 1” and was a target compound identified only through site-specific information [14]. This challenge would be difficult for *de novo* structure elucidation, as it is quite a big molecule and has a wide variety of functional groups. The many functional groups also make it difficult to incorporate predictive selection strategies. Even with knowledge of the true structure, it was difficult to annotate all the major MS/MS peaks using either simple bond-breaking approaches or the general and library fragmentation rules in Mass Frontier [5]. The major annotations are shown in Figure A9. It is likely that a much more detailed elucidation of the fragmentation processes would be needed to annotate all peaks, which was beyond the scope of this article. 

**Figure A10:**
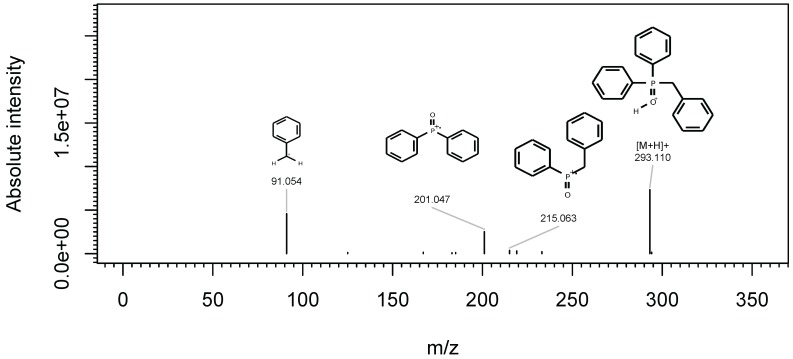
Challenge 13: annotated merged MS/MS spectra of benzyldiphenylphosphine oxide (ESI, positive mode).

**Challenge 13** was benzyldiphenylphosphine oxide and was one of the easier challenges, for database searching and structure generation alike, when taking the spectrum into account. The only “degree of freedom” was the location of the CH_2_ or CH_3_ group (*i.e.*, whether a benzyl or methylphenyl substituent was present). The spectrum of this compound and similar compounds were uploaded to MassBank [8,17] before the submission deadline. The major fragments are shown in Figure A10. 

**Figure A11:**
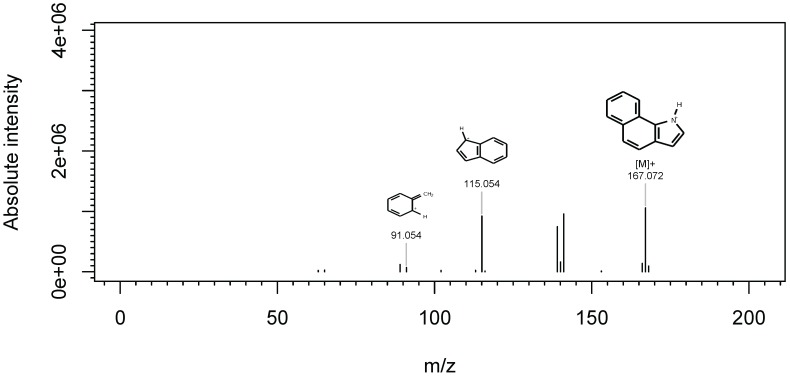
Challenge 14: annotated merged MS/MS spectra of 1H-benz[g]indole (APCI, positive mode).

**Challenge 14** was 1H-benz[g]indole, another stable, aromatic compound. Although the spectra (shown together in Figure A11) display more fragments than Challenge 11, the collision energy is much greater here (HCD 120 and 180 NCE, compared with CID at 35 NCE above). The fragments at *m*/*z* 167 and *m*/*z* 168 are potentially a mix of [M]^+^^·^ and [M + H]^+^, with a H loss as the first major fragment. The remaining fragments are successive two-member losses from the aromatic system; first, CNH followed by two C_2_H_x_ losses. The fragments given in Figure A11 are indicative; a rearrangement may stabilize the fragments at *m*/*z* 141 and *m*/*z* 91. 

**Figure A12:**
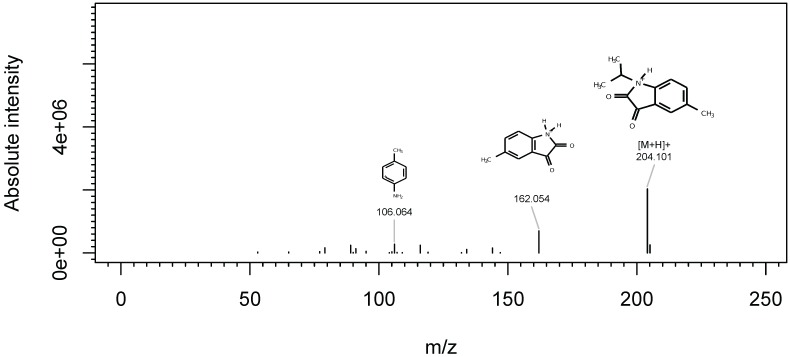
Challenge 15: annotated merged MS and MS/MS spectra of 1-isopropyl-5-methyl-1H-indole-2,3-dione (APCI, positive mode).

**Challenge 15** was 1-isopropyl-5-methyl-1H-indole-2,3-dione and has quite a small, aromatic-stabilized system with a distinctive isopropyl loss in the MS/MS spectrum, followed again by the break-up of the aromatic system (see Figure A12). The presence of *m*/*z* 91 indicates that the methyl group is attached to a benzene ring; *m*/*z* 106 indicates also that the N is attached to the same benzene ring. The carbonyl groups again display a loss of water, as well as the full substituent. 

**Figure A13:**
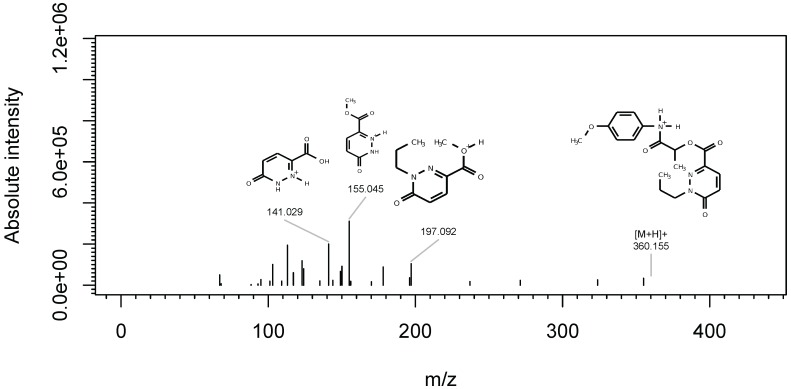
Challenge 16: annotated merged MS and MS/MS spectra of [1-(4-methoxyanilino)-1-oxopropan-2-yl] 6-oxo-1-propylpyridazine-3-carboxylate (APCI, positive mode). The [M+H]+ ion was not observed in the measured spectra.

**Challenge 16** was [1-(4-methoxyanilino)-1-oxopropan-2-yl] 6-oxo-1-propylpyridazine-3-carboxylate. This challenge was a candidate for an unknown identification, where the original unknown remains unidentified. This compound experiences significant fragmentation, such that neither the molecular ion nor any adducts of the molecular ion are present in the MS. The MS and MS/MS are merged in the spectrum displayed in Figure A13. Energy-based fragmentation scoring (as in, e.g., MetFrag [18]) can prioritize the wrong compounds, such as here, where the fragmentation was too favorable. Thus, the presence in a compound database does not necessarily mean that the compound is conducive to identification via MS/MS analysis. 

**Figure A14:**
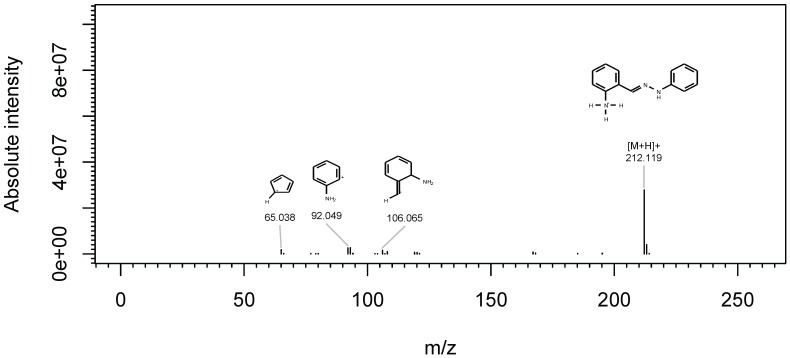
Challenge 17: annotated merged MS and MS/MS spectra of nitrin (ESI, positive mode).

**Challenge 17** is nitrin, another unconfirmed tentative identification where the presence of a C_6_H_5_(N ≡ N)^+^ fragment in the original unknown spectrum led to the (incorrect) tentative identification of nitrin. The peak instead arose from a nitrogen adduct formed during MS/MS measurements, a phenomenon observed with several aromatic compounds (e.g., [15,19]). One result of the adduct detection was the expansion of the fragment formula annotation option in RMassBank to include adducts by adding N_2_ and O to the allowed elements of the subformulas [15]. The spectrum of Challenge 17 is shown in Figure A14. The fragment at *m*/*z* 105.044 corresponding to a C_6_H_5_(N ≡ N)^+^ fragment is conspicuously absent in the MS/MS spectrum of this compound. Instead, fragmentation occurs between the Ns, and only a few pieces of the molecule are observed. Interestingly, the fragment at *m*/*z* 77 (characteristic for a phenyl substituent) was very small, confirming that fragmentation occurs preferably between the Ns. 

## 3. GC-MS Challenges and Solutions (Category 3 and 4)

All GC-MS challenges are summarized in Table 2 and were sourced from real environmental samples and were confirmed with reference standards. This requirement of being certain of the identity (for the purpose of a contest), but also not being too easy to find in a database, was a big challenge for the GC-MS data, as over 200,000 compounds are now included in GC-MS databases, such as NIST [2]. As a result, challenges were selected where the probability for a database match was relatively low, *i.e.*, not a ‘straightforward’ identification. Many of these are quite standard compounds, but the spectra were taken from real samples (instead of the database) to add some variety. A couple of isomers were chosen to see if computational methods could match the ability of databases to distinguish isomers. A couple of challenges that did not meet this ‘low probability’ requirement were added to diversify the challenge set further. There were a lot more halogens (chlorine only) present in these spectra compared with the LC-HRMS challenges. 

**Figure C18:**
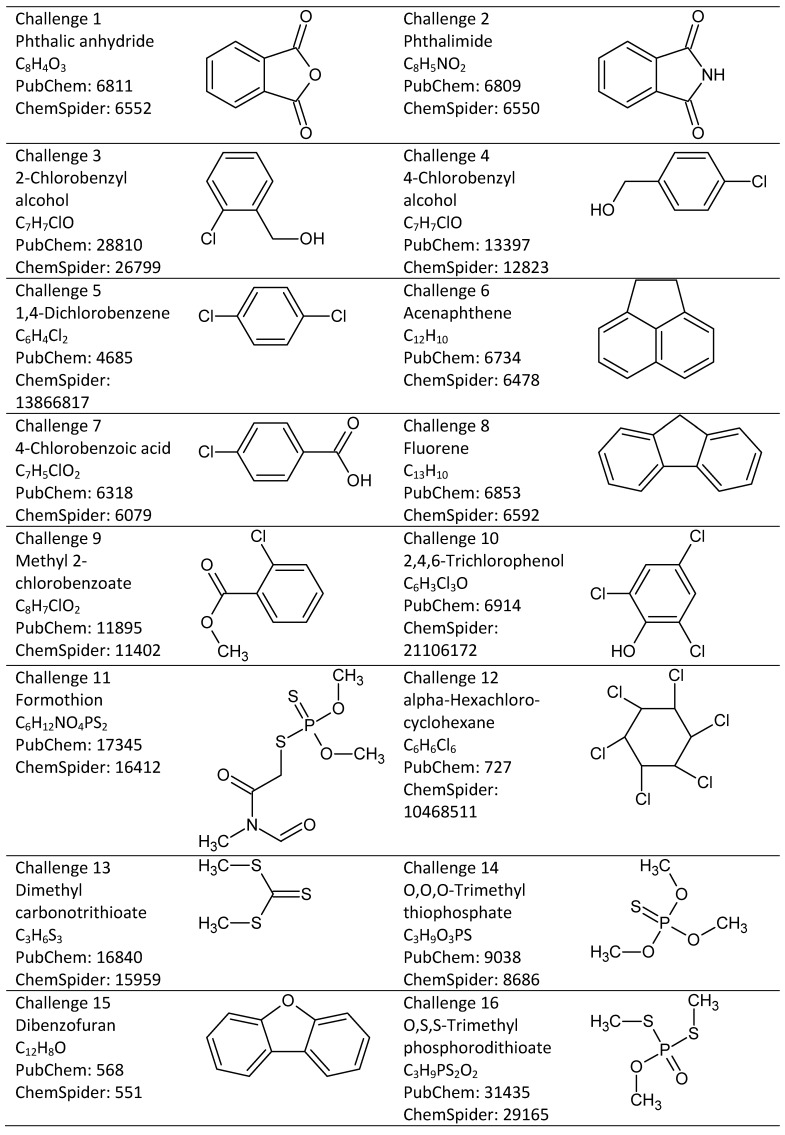
Structures and identifiers for GC-MS Challenges 1–16.

As no external participants participated in these categories, these challenges are not described in detail. The structures and several identifiers are given in Appendix C, Figure C18. 

**Table 2:** Gas chromatography (GC) Challenges for CASMI 2012.

Challenge	Trivial Name	Formula	Exact mass
1	Phthalic anhydride	C_8_H_4_O_3_	148
2	Phthalimide	C_8_H_5_NO_2_	147
3	2-Chlorobenzyl alcohol	C_7_H_7_C_l_O	142
4	4-Chlorobenzyl alcohol	C_7_H_7_C_l_O	142
5	1,4-Dichlorobenzene	C_6_H_4_C_l2_	146
6	Acenaphthene	C_12_H_10_	154
7	4-Chlorobenzoic acid	C_7_H_5_C_l_O_2_	156
8	Fluorene	C_13_H_10_	166
9	Methyl 2-chlorobenzoate	C_8_H_7_C_l_O_2_	170
10	2,4,6-Trichlorophenol	C_6_H_3_C_l3_O	196
11	Formothion	C_6_H_12_NO_4_PS_2_	257
12	alpha-Hexachlorocyclohexane	C_6_H_6_C_l6_	290
13	Dimethyl carbonotrithioate	C_3_H_6_S_3_	138
14	O,O,O-Trimethyl thiophosphate	C_3_H_9_O_3_PS	156
15	Dibenzofuran	C_12_H_8_O	168
16	O,S,S-Trimethyl phosphorodithioate	C_3_H_9_PS_2_O_2_	172

### 3.1. GC-MS Challenges 1 and 2

These two challenges were chosen due to the availability of standards for retention index (RI) calculation [20]. These were very closely related; only an O and NH are different. 

### 3.2. GC-MS Challenges 3 to 16

Challenges 3–16 came from the EDA of a groundwater sample from Bitterfeld, Germany [21,22]. Fractionation using reverse-phase high performance liquid chromatography (RP-HPLC) with a C18 column and preparative GC (pcGC) was performed prior to the final GC-MS analysis (for more details, see [21]). As a result, partitioning information could be calculated for the individual fractions, and this provided additional information for the identification, which was made available to the CASMI participants. The compounds identified in the sample are quite common environmental contaminants that could have resulted in almost trivial identification results for participants with access to a large GC-MS database. 

## 4. Recommendation for Future CASMIs

The problem of insufficient spectra and, especially for GC, too many spectra in the databases, could be improved in future CASMIs by sourcing compounds from a synthetic laboratory, which would be able to provide rare compounds, but also confirm their identity. ‘Unknown unknowns’ are not suitable for a competition, as the identity must be known to declare the winner(s). 

Due to the lack of participants in the GC-MS categories, the organizers of the next CASMI may consider adding a different category to complement the accurate mass LC-HRMS categories. Some possibilities include an accurate mass GC category, or GCxGC-TOF, or changing the focus to different MS/MS ionization techniques, rather than forming distinct GC and LC categories. It is also plausible that only two categories should be offered in the next CASMI, *i.e.*, restricting the competition to Categories 1 and 2 only. Another enhancement to the LC-HRMS categories could be the inclusion of challenges measured along with a set of standard compounds to provide reference retention times, or providing participants with candidate lists that they would need to rank. 

One way to improve participation in future CASMIs could be to provide additional incentives, such as prizes. The opportunity to submit papers to a special issue does not appear to have been sufficient incentive to attract many participants in the 2012 contest. Although sponsorship would be an option, it can compromise the independence (or at least, the appearance of independence) of the competition. An alternative, more scientific incentive could be the organization of a CASMI identification workshop, which would require more participants to be successful. 

CASMI could also provide the ideal exchange platform for selected ‘unknown unknowns’ in the future, where scientists could submit their unknowns and offer other experts (and expert systems) the chance to identify them. Obviously, no winners can be declared when the answer is unknown; the contributor of the ‘unknown unknown’ would be required to decide the appropriate ‘reward’. 

## Figures and Tables

**Figure A7:**
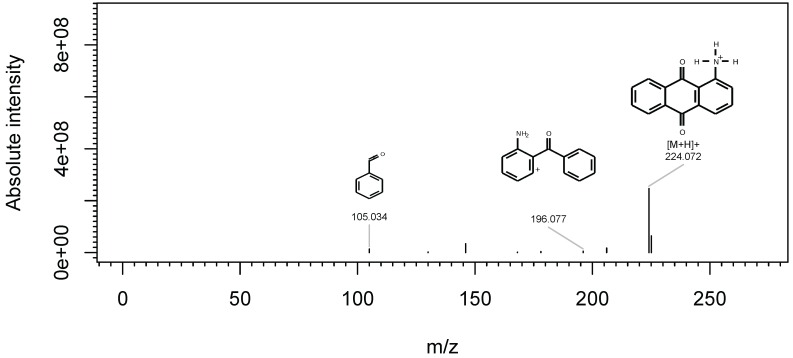
Challenge 10: annotated MS/MS spectrum of 1-aminoanthraquinone (ESI, positive mode).

**Figure B15:**
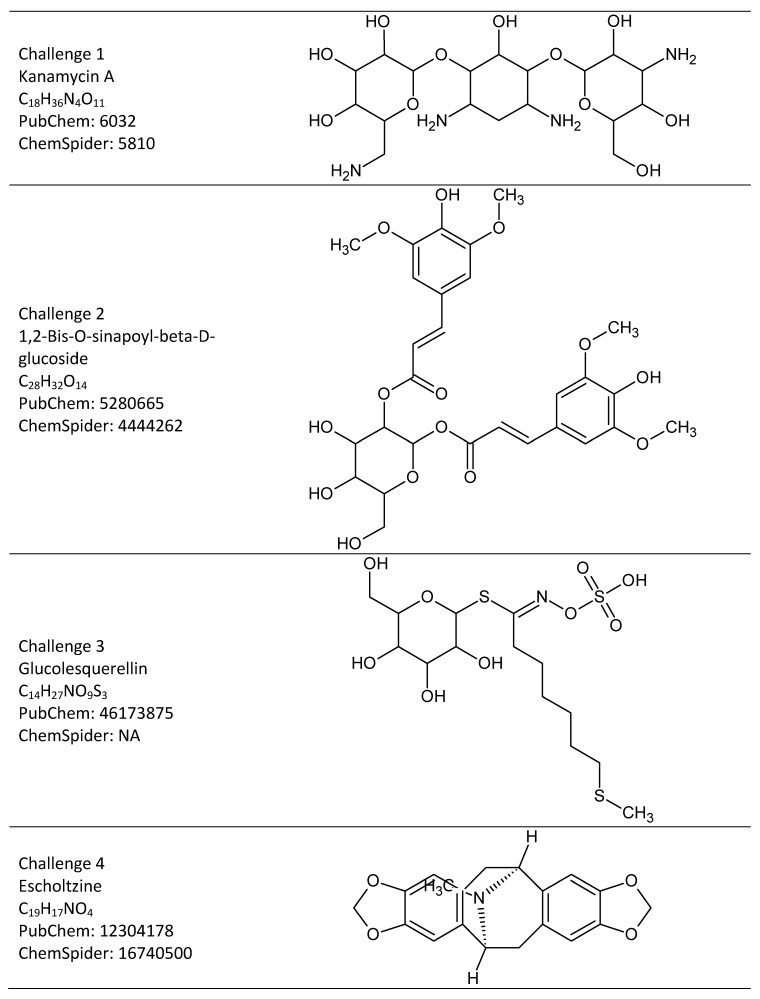
Structures and identifiers for LC-HRMS Challenges 1–4.

**Figure B16:**
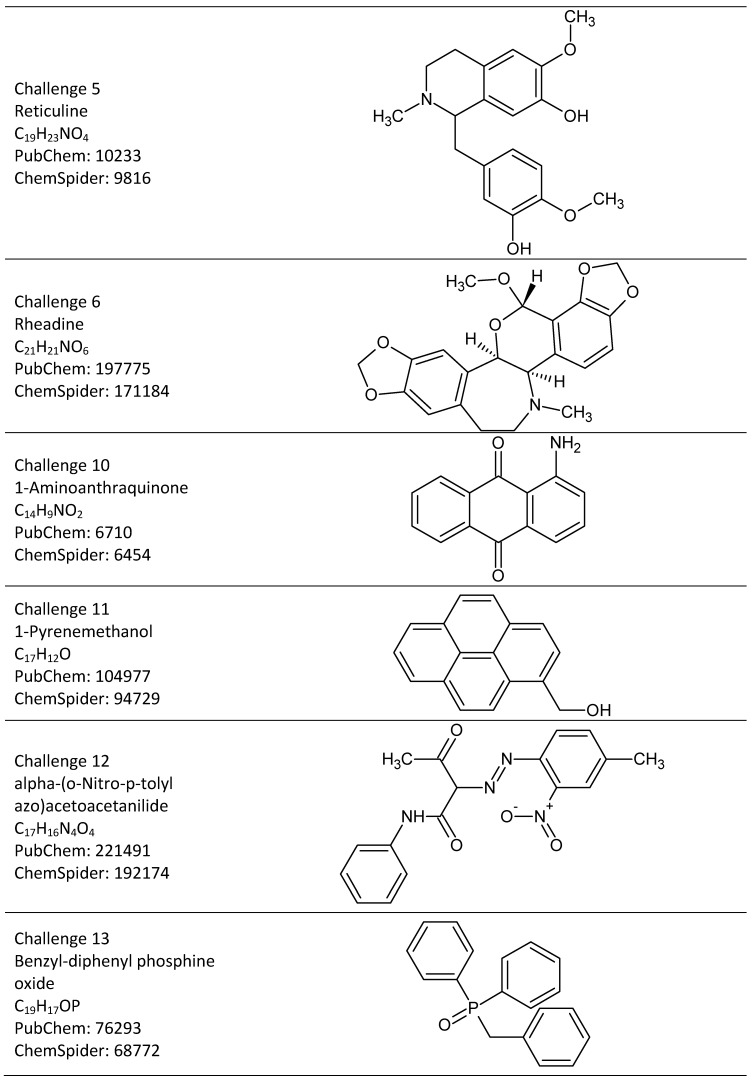
Structures and identifiers for LC-HRMS Challenges 5–13.

**Figure B17:**
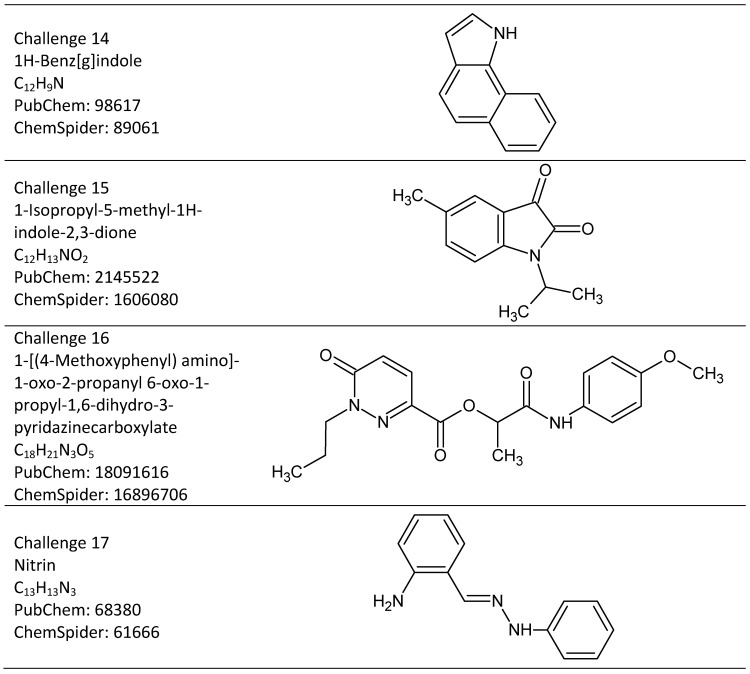
Structures and identifiers for LC-HRMS Challenges 14–17.

## Appendix

### A. Annotated spectra

This appendix contains the annotated spectra for LC-MS Challenges 1–17. The structures were determined with the help of experience, ChemSketch [4] and MassFrontier [5]. Selected fragments were added to a script in R [7], while the processing of the spectra, including placement of the fragments, was automatic. OpenBabel [6] was used to generate the images.

### B. Structures for the LC-HRMS challenges (Categories 1 and 2)

Figure B15, Figure B16 and Figure B17, contain the structures and identifiers for the LC-HRMS challenges, Categories 1 and 2.

### C. Structures for GC-MS Challenges

Figure C18 contains the structures and identifiers for the GC-MS challenges, Categories 3 and 4.
